# Harnessing nanomedicine to overcome the immunosuppressive tumor microenvironment

**DOI:** 10.1038/s41401-020-0424-4

**Published:** 2020-05-18

**Authors:** Bo Sun, Hyesun Hyun, Lian-tao Li, Andrew Z Wang

**Affiliations:** 1 Department of Pharmacology and Toxicology, College of Pharmacy, University of Arizona, Tucson, AZ 85721 USA; 2 Laboratory of Nano and Translational Medicine, Carolina Center for Cancer Nanotechnology Excellence, Carolina Institute of Nanomedicine, University of North Carolina at Chapel Hill, Chapel Hill, NC 27599 USA; 3 Department of Radiation Oncology, Lineberger Comprehensive Cancer Center, University of North Carolina at Chapel Hill, Chapel Hill, NC 27599 USA; 4 Cancer Institute, Xuzhou Medical University, Xuzhou, 221004 China; 5 Department of Radiation Oncology, Affiliated Hospital of Xuzhou Medical University, Xuzhou, 221004 China

**Keywords:** cancer immunotherapy, tumor microenvironment, immunosuppression, immune cells, cytokines, enzymes, nanomedicine

## Abstract

Cancer immunotherapy has received extensive attention due to its ability to activate the innate or adaptive immune systems of patients to combat tumors. Despite a few clinical successes, further endeavors are still needed to tackle unresolved issues, including limited response rates, development of resistance, and immune-related toxicities. Accumulating evidence has pinpointed the tumor microenvironment (TME) as one of the major obstacles in cancer immunotherapy due to its detrimental impacts on tumor-infiltrating immune cells. Nanomedicine has been battling with the TME in the past several decades, and the experience obtained could be exploited to improve current paradigms of immunotherapy. Here, we discuss the metabolic features of the TME and its influence on different types of immune cells. The recent progress in nanoenabled cancer immunotherapy has been summarized with a highlight on the modulation of immune cells, tumor stroma, cytokines and enzymes to reverse the immunosuppressive TME.

## Introduction

**Fig. 1:**
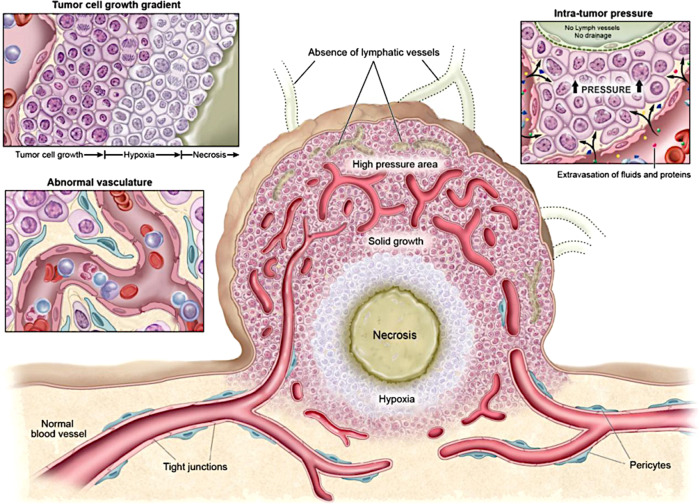
Physiological characteristics of tumor tissue and vasculatures. Adapted from REF [13] with permission by Theranostics under Creative Commons Attribution (CC BY-NC) License.

The tumor microenvironment (TME) is a complex ecosystem consisting of not only tumor cells but also vasculature, stroma, infiltrating immune cells, fibroblasts, and other noncellular tissues [1, 2]. Tumor vasculature has striking differences from vessel networks in normal tissues. Leaky vasculature is malformed under elevated levels of vascular endothelial growth factor (VEGF), which is secreted by fast-growing tumor cells [3]. The disorganized patterns and abnormal diameters of tumor vessels result in nonuniform oxygen and nutrient supplies for cancer cells [4, 5]. Hypoxia has been found in more than 50% of solid tumors because the oxygen supply from aberrant tumor vasculature cannot meet the need for rapid tumor development [6]. Furthermore, tumors adopt aerobic glycolysis as a main source of ATP to feed rapidly proliferating cells [7]. Acidic products from aerobic glycolysis, such as lactate, contribute to the lower pH in the extracellular matrix (ECM) of tumors than in normal tissues [8, 9]. Hypoxia-inducible factor (HIF-1) can aggravate acidosis by upregulating glycolytic enzymes, glucose transporters and lactate production [10]. High interstitial fluid pressure (IFP) is another important physiological parameter of the TME. Compression from the increasing tumor mass, hyperpermeable vascular walls and the absence of functional lymphatic vessels contributes to elevated fluid pressure within tumors [11, 12]. Hypoxia, acidosis, and IFP fortify the TME against the entry of therapeutics and immune attack (Fig. 1) [13].

## The interplay between immune cells and the TME

**Fig. 2:**
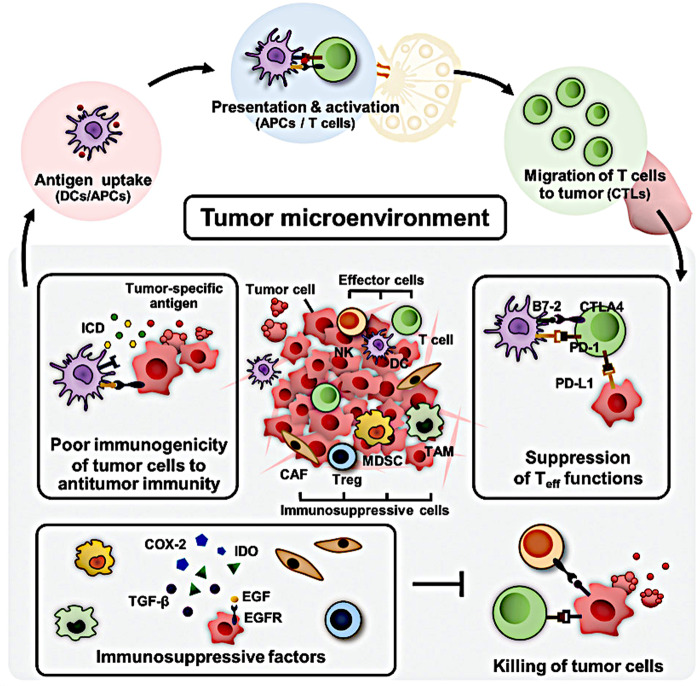
Immune cells in the immunosuppressive TME. DCs/APCs: dendritic cells/antigen-presenting cells, CTLs: cytotoxic T lymphocytes, ICD: immunogenic cell death, NK: natural killer cells, CAF: cancer-associated fibroblasts, Treg: regulatory T-cells, MDSC: myeloid-derived suppressor cells, TAM: tumor-associated macrophages, T_eff_ : effector T-cells, COX-2: cyclooxygenase-2, IDO: indoleamine 2,3-dioxygenase, TGF-β: transforming growth factor-β, EGFR: epithelial growth factor receptor. Adapted from REF [167] with permission by WILEY-VCH.

Growing evidence has underscored the significant roles of immune cells in different phases of tumor progression. Major immune cells in the TME include macrophages, dendritic cells (DCs), neutrophils, monocytes, myeloid-derived suppressor cells (MDSCs), natural killer (NK) cells, T cells, and B cells (Fig. 2) [14, 15]. The interplay between immune cells and other cellular and noncellular components in the TME largely determines disease progression and therapeutic outcomes [16]. In addition to the aforementioned cardinal features of the TME, altered tumor metabolism is detrimental to the activation of immune cells and subsequent differentiation and memory formation [17]. In addition to hypoxia, glucose depletion and lactate production, amino acid depletion and increased lipid metabolism also play critical roles in the inhibition of effector cells, induction of regulatory/suppressor cells and upregulation of programmed cell death-ligand-1 (PD-L1) in the TME [18, 19].

Similar to cancer cells, activated T lymphocytes upregulate aerobic glycolysis and glutamine metabolism to facilitate the proliferation and differentiation of effector T cells (T-effs) [20]. Tumor cells compete with T cells for nutrients in the TME due to their similar metabolic processes, resulting in compromised T-cell receptor (TCR) signaling and dampened production of cytokines such as interferon-γ (IFN-γ), interleukin-2 (IL-2), and tumor necrosis factor-α (TNF-α) [21–23]. Furthermore, exhausted T cells are also characterized by high expression of several immune checkpoint receptors, such as PD-1, cytotoxic T-lymphocyte-associated protein 4 (CTLA-4), T-cell immunoglobulin and mucin-domain containing-3 (TIM-3) and lymphocyte activation gene-3 (LAG-3), which lead to impaired T-cell functions [24, 25]. The immunosuppressive TME also affects the functions of NK cells, another major player in antitumor immunity whose tumoricidal effect is manipulated by a balance between inhibitory and activating signaling after priming by DCs, macrophages, and/or interleukins [26–28]. Glucose depletion represses the cytotoxic activity and cytokine production of NK cells in the TME [29, 30]. Accumulated metabolites in the TME, such as adenosine and lactate, also suppress the functions and survival of NK and T cells [31, 32]. The limited availability of arginine, leucine, and glutamine has a direct impact on mammalian target of rapamycin complex 1 and c-Myc signaling, which control the differentiation and functions of NK and T cells [33, 34]. In addition, the proliferation and cytokine production of NK cells can be inhibited by increased catabolites of amino acids that are mediated by upregulated enzymes, such as indoleamine 2,3-dioxygenase (IDO), arginase, and inducible nitric oxide synthase (iNOS) [35–38].

Among these tumor-infiltrated immune cells, tumor-associated macrophages (TAMs) are probably the largest population in the TME [2, 39]. TAMs not only coexist but also coevolve with tumor cells. In response to tumor progression, M2-like TAMs result from interactions between tumor cells and M1-like TAMs that are predominant in very early oncogenesis [40, 41]. Under the influence of neighboring cancer cells, M2-like TAMs foster malignancy by releasing cytokines, such as IL-6, TNF-α and C–C chemokine ligands, promoting aerobic glycolysis and hypoxia [42, 43]. M2-polarized TAMs also dysregulate immune responses by promoting PD-L1 expression, remodeling the ECM to trap infiltrating T cells, and producing phagocytosis-inhibiting proteins and enzymes that favor metabolite accumulation in the TME [44–46].

Hypoxia has complex roles in the immunosuppressive TME. Hypoxia induces tumor cells to secrete immunosuppressive molecules, such as transforming growth factor-β, VEGF, IL-10, CC-chemokine ligands, galectins, and COX-2, contributing to the generation and accumulation of M2-polarized TAMs, regulatory T cells and MDSCs, which suppress DCs and T cells in the TME and negatively regulate tumor antigen presentation [47]. Hypoxia also has a direct impact on antitumor effector cells. Recent studies have shown that hypoxia does not compromise the cytolytic capacities of cytotoxic T lymphocytes (CTLs) but restricts the number of CTLs in the TME to control tumor growth [48]. Interestingly, a few studies suggested a positive role of hypoxia in CTLs. Hypoxia stimulates the upregulation of 4-1BB on the surface of activated T cells, which could potentially benefit anti-4-1BB agonist therapy [49, 50]. The function of NK cells is partially inhibited by hypoxia. NK cells fail to upregulate surface expression of several activating receptors under hypoxia, but antibody-dependent cellular cytotoxicity is not affected [51, 52]. The influence of hypoxia on T and NK cells is still unclear and warrants further study.

In recent decades, many therapeutic strategies have been developed to target different aspects of the TME. As one promising strategy, nanomedicine represents a versatile platform that exploits nanoparticles (NPs), which are fine-tuned nanoscale materials for drug delivery and diagnosis [53]. The physicochemical properties of NPs, such as size, shape, and surface charge, can be tailored to perform controlled release of payloads, passively accumulate at tumors by enhanced permeability and retention effects, or specifically target tumors [54]. NPs incorporated with physical and biological technologies have already been utilized in the delivery of vaccine adjuvants, cytokines, and immune checkpoint blockades (ICBs) to modulate the TME (Table 1), with the aim of improving the outcomes of current radiotherapy, chemotherapy, and immunotherapy [55]. Herein, we discuss the immunometabolism of major immune cells in the TME and summarize the recent progress in nanomedicine that reprograms these cells in an immunosuppressive context, which represents new strategies for the development of next-generation immunotherapies.

**Table 1:** A summary of immunomodulatory approaches in the TME.

Immunomodulatory approach		Mechanism	Delivery strategy	Example
Physical approaches	Radiotherapy	Radiation-induced DNA damage results in cell death and potentiates antitumor immune responses [168].	Radiotherapy combined with immune adjuvant or checkpoint regulator to promote immunological response and antigen presentation.	Nanovaccine [169, 170] Anti-PD-1 and anti-OX40 conjugated nanoparticle [61]
	Photodynamic therapy	Reactive oxygen species generated by photochemical reaction causes cell death, provoking antitumor immunity [171].	Photodynamic therapy combined with immune adjuvant or checkpoint regulator to promote immunological response and antigen presentation.	Anti-PD-L1 [172, 173] Anti-PD-1 [174] PD-L1 silencing siRNA [175] CpG [176, 177]
	Hyperthermia therapy	High temperature (40–45 °C) generated locally by light, magnetic field, radiation or microwave causes tumor cell death, provoking antitumor immunity [178].	Hyperthermia therapy combined with immune adjuvant, checkpoint regulator, or CAR-T therapy.	Anti-PD-L1 [179, 180], anti-PD-1 [181–183]; poly (I:C) [184], CpG [185, 186], LPS, GM-CSF, and anti-PD-1 [187]; CAR-T [188]
	Electrotherapy: Electrochemotherapy Tumor-treating fields	Local electroporation-facilitated drug uptake results in tumor cell death and release of tumor-associated antigens, activating immune reaction [189]; Mitotic disruption caused by alternating electric fields potentiates immunogenic cell death [190].	Electrochemotherapy combined with immune checkpoint blockade; Tumor-treating fields combined with immune checkpoint blockade.	Anti-CTLA-4 [191], anti-PD-1 and anti-CTLA-4 [192] Anti-PD-1 [193]
Biological approaches	Oncolytic viruses	Virus replication in selectively infected tumor cells induces immunogenic cell death and hence stimulates the immune system [194].	Intratumoral, intravenous or intraperitoneal administration of oncolytic viruses armed with genes encoding immunostimulatory molecules [195, 196].	New castle disease virus expressing anti-CTLA-4 scFv [197] Adenovirus expressing GM-CSF or anti-CTLA-4 [198] Adenovirus expressing IL-2 and TNF-α [199]
	Cytokines (GM-CSF, FLT3L, IL-2, IL-12, etc. and TGF-β inhibition)	GM-CSF and FLT3L enhance DCs mobilization and recruitment in TME; IL-2, IL-12, etc. modulate the differentiation/activation and expansion/survival of T cells in TME [148]; TGF-β inhibition attenuates the suppression on DCs, NK cells, and T cells in TME [200].	Intra or peri-tumoral delivery of free or particle-encapsulated cytokines. Targeted delivery of small molecular TGF-β inhibitor or antibody against TGF-β receptor.	GM-CSF in hydrogel [201–203]; FLT3L [204–206]; More examples of cytokine-loaded liposomes or polymeric particles can be found in a review [148] TGF-β inhibitor-loaded nanoparticles targeting T cells [207]; Bifunctional fusion protein targeting PD-L1 and TGF-β receptor II [208]
	Immune checkpoint regulators (blockade or agonist)	Immune checkpoint blockade, such as anti-PD-1, anti-PD-L1, and anti-CTLA-4, enhance the function and survival of T cells; Agonists, such as anti-CD40, anti-OX40, and anti-4-1BB, facilitate the activation of APCs and T cells.	Targeted nano-delivery system of immune checkpoint regulator.	Nanoscale immunoconjugates with anti-PD-1 or anti-CTLA-4 [63]; nanoparticles of PD-1/PD-L1 inhibitor [209]; 4-1BB-agonistic trimerbody [210]; Carbon nanotubes loaded with CpG and anti-CD40 [211]
	Adjuvants (TLRs agonists, STING agonists, etc.)	Adjuvants facilitate the presentation of tumor-associated antigens and the production of immunoregulatory molecules [212, 213].	Intratumoral delivery of encapsulated adjuvants.	Nanoparticle-conjugated TLR7/8 agonist combined with anti-PDL1 and Flt3L [206]; High-density lipoprotein nanodisc loaded with CpG [214]; Cytotoxic cationic silica nanoparticles complexed with c-di-GMP [215]

Adapted from REF [55] with permission by Frontiers in Immunology under Creative Commons Attribution License (CC BY)

## Modulation of immune cells in the TME

### Modulation of T-effs

Metabolic reprogramming in T cells is triggered by antigen recognition by the TCR in the presence of costimulatory factors. Aerobic glycolysis is adopted to provide energy and nutrients more efficiently during T-cell activation than oxidative phosphorylation (OXPHOS) [21]. PI3K/Akt/mTOR and c-Myc are two key signaling pathways that elevate the expression of glucose transporter-1, which facilitates glycolysis in T-effs [56]. However, these two pathways could be compromised by reduced glutamine and leucine metabolism in the TME, resulting in hampered T-cell activation [57]. Lipid metabolism also plays an essential role in the functions of T-effs. An increased level of cholesterol was found in activated CD8^+^ T cells, which promotes TCR clustering and signaling [58]. Enhanced fatty acid catabolism would be helpful for maintaining the function of CD8^+^ T cells in the malnourished TME [59]. For example, acetyl-CoA acetyltransferase-1 (ACAT-1) is a cholesterol esterification enzyme expressed in CD8^+^ T cells that downregulates T-cell activation by reducing free cholesterol levels [58]. In an effort to potentiate CD8^+^ T cells, avasimibe, an ACAT-1 inhibitor, was combined with nanoliposomes containing paclitaxel and the immunoadjuvant α-GalCer for chemoimmunotherapy of a melanoma model [60]. Avasimibe strengthened the cytotoxicity of CD8^+^ T cells by inhibiting ACAT-1 and increasing free cholesterol in the plasma membrane. This combined therapy significantly suppressed tumor growth and prolonged the survival of mice with melanoma compared with those treated with any single therapy.

Immune checkpoint signaling has significant impacts on the glucose metabolism of tumor cells and T cells. Antibodies or antibody-conjugated NPs that bind PD-1/PD-L1, 4-1BB, or CTLA-4 can restore glucose levels in the TME by regulating the Akt/mTOR or liver kinase B1/AMP-activated protein kinase/acetyl-CoA carboxylase signaling pathway, allowing T-cell glycolysis and cytokine production [22, 61, 62]. Galstyan et al. developed poly (β-*L*-malic acid) nanoconjugates to facilitate delivery of checkpoint inhibitory antibodies to brain glioma across the blood brain barrier (BBB) [63]. Anti-CTLA-4 or anti-PD-1 were covalently conjugated to the polymer backbone, and BBB crossing was achieved with transferrin receptor antibodies. An increase in CD8^+^ T cells was observed in the interstitium of intracranial GL261 glioblastoma treated with a combination of two checkpoint inhibitory antibody-nanoscale immunoconjugates (NICs). The survival of tumor-bearing mice was markedly prolonged when treated with the NIC combination in comparison with free antibodies or single antibody NIC. This study underscored the potential of polymer-based trans-BBB delivery to improve the local immune response to brain tumors.

CD4^+^ T cells can differentiate into a variety of subtypes and thus coordinate a wide range of immune responses in autoimmune diseases, inflammatory diseases, and cancers [64, 65]. Generally, CD4^+^ T cells can be divided into two subsets, T helper 1 (Th1), and T helper 2 (Th2), which elicit antitumor effects and tumor-promoting effects, respectively. Emerging subsets, such as immunosuppressive regulatory T cells, follicular helper T cells, and Th9, Th17 and Th22 cells, have also been classified [66–68]. Outstanding examples of nanomedicine specifically modulating CD4^+^ T cells have not been adequately reported, probably due to the diversity of the CD4^+^ population and their plasticity in response to different immune microenvironments [69].

**Fig. 3:**
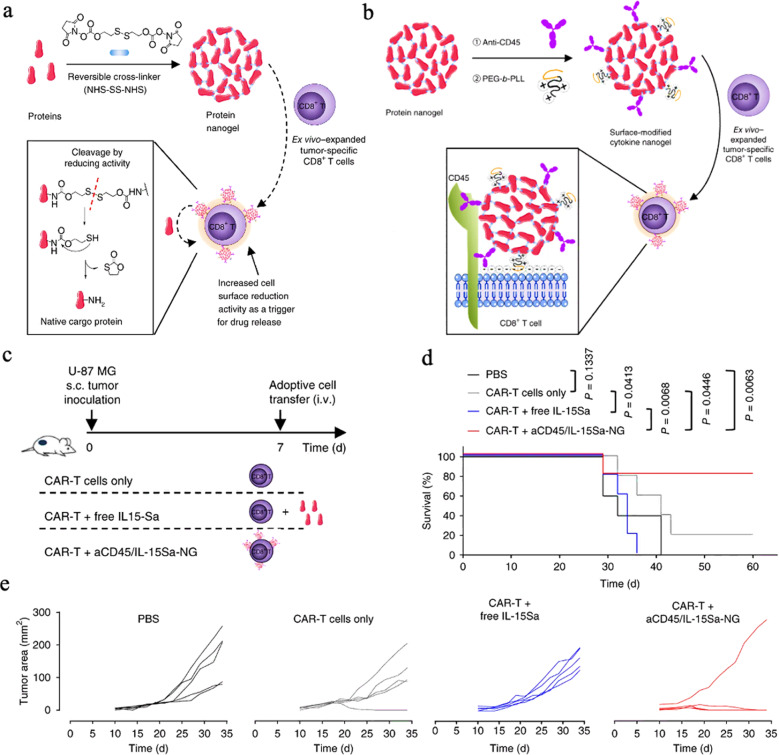
**a** Scheme for protein nanogel synthesis and for release of protein in response to reducing activity in the local microenvironment. **b** Scheme for surface modification of cytokine-nanogels to facilitate efficient and stable anchoring on T-cell surface. **c** Experimental scheme. Luciferase-expressing U-87 MG human glioblastoma cells (1.0 × 10^6^) were subcutaneously injected into NSG mice (*n* = 5 mice/group). Mice received i.v. adoptive transfer of human T cells (2.6 × 10^6^ total cells, 38% transduced with EGFR-targeting CAR (1.0 × 10^6^ CAR-T cells)) on day 7. Mice were treated with sham saline injections, CAR-T cells alone, CAR-T cells followed by 13.8 µg of free IL-15Sa, or CAR-T cells coupled with aCD45/IL-15Sa-nanogels (13.8 µg). **d** Survival curves of treatment groups. **e** Individual tumor growth curves. Statistical analyses were performed by using two-way ANOVA test for tumor growth data and log-rank test for survival curves. Data represent the mean ± SEM. All data are one representative of at least two independent experiments. Adapted from REF [71] with permission by Springer Nature.

Targeted T-cell therapies have demonstrated great promise in the treatment of blood cancer. In 2017–2018, two T-cell therapies, Yescarta ^TM^, and Kymriah ^TM^, were approved by the FDA for adult patients suffering from relapsed or refractory lymphoma and leukemia [70]. However, applying the same paradigm in solid malignancies may not be an easy task. Malformed vasculature and a nutrient-depleted TME represent one of the major hurdles between antigen-specific T cells and tumors. To maintain the viability of tumor-specific T cells, supportive NPs filled with interleukin-15 super-agonist complex (IL-15Sa) were attached to the T-cell surface via anti-CD45 antibodies (Fig. 3) [71]. IL-15Sa release was initiated upon T-cell activation. Tumor growth was significantly suppressed after multiple administrations of T cells equipped with cytokine backpacks without inducing dose-limited toxicity compared with that of mice that received equivalent doses of T-cell transfer alone or T-cell transfer plus free cytokines. In another effort to create a stimulatory tumor milieu for subsequent T-cell infusion, a PI3K inhibitor (PI-3065) and invariant natural killer T-cell (iNKT) activator (7DW8-5) were codelivered in liposomes decorated with tumor-targeting iRGD peptides [72]. Immune suppressing cells were reduced more than fourfold in the 4T1 tumor milieu after treatment with multiple doses of dual-drug liposomes in contrast to those treated with empty vehicles. This TME priming strategy augmented the accumulation of T-effs, such as CD8^+^ T cells and iNKT, in tumors and thus boosted the therapeutic effect of subsequent T-cell therapy.

### Modulation of regulatory T cells (T-regs)

In contrast to T-effs, T-regs play an essential role in the immune escape of tumors, and the TME favors T-reg recruitment and differentiation [47, 73]. A marked increase in T-regs in the TME has been recognized as a hallmark of many solid malignancies. Under hypoxia, HIF-1α in T-regs enhances the migration and suppressive effects of T-regs in the TME [74]. The proliferation and function of T-regs are supported by their flexibility in shifting metabolic processes to survive in a glucose-restricted but lactate-abundant setting in comparison with rigid T-effs. Several studies have shown that T-regs have a stronger ability to compete for glucose and execute glycolysis than T-effs, which leads to T-cell exhaustion in cooperation with tumor cells [20, 75, 76]. In addition to glucose competition, T-regs take up and utilize accumulated fatty acids in the TME via fatty acid oxidation (FAO) to fuel and modulate their expansion and inhibitory activities [77, 78]. Moreover, T-regs suppress activated T cells and APCs by releasing inhibitory molecules, including IL-2, 4, 6, TGF-β, granzymes, and perforin [79, 80].

Recent studies have highlighted the crucial roles of glucocorticoid-induced tumor necrosis factor receptor-related protein (GITR, CD357) in activated T cells and T-regs [81]. Agonistic antibodies targeting GITR exert evident therapeutic effects by depleting T-regs and reinvigorating CD8^+^ T cells by reducing PD-1 and LAG-3 expression [82]. However, anti-GITR monotherapy may be inadequate to achieve significantly improved clinical responses [81]. Anti-GITR therapy was combined with anti-PD1 therapy to attenuate the immunosuppression of T-regs induced by radiation therapy in an anti-PD1-resistant preclinical tumor model, yielding improved survival and tumor eradication [83]. In addition to agonistic monoclonal antibodies, the small molecular drug imatinib, an inhibitor of T-regs and IDO pathway in tumor cells [84, 85], was combined with photodynamic/thermal therapy for photoimmunotherapy in a preclinical model of melanoma [86]. Imatinib and the near-infrared (NIR) photosensitizer IR-780 were coencapsulated in PLGA hybrid nanoparticles (hNPs) with hybrid surface layers for pH-triggered release of payloads in the acidic TME. Tumor ablation was observed in the mice that were treated with hNPs plus NIR irradiation, and 80% of them survived until the end of the efficacy study (60 days), while mice that were treated with PBS, free imatinib or free IR-780 hardly lived for 50 days. Immunohistochemical evaluation and flow cytometry results demonstrated an increased population of CD4^+^ and CD8^+^ T cells and a reduction in T-regs in the TME of mice treated with hNPs plus photoinduced therapy, which supports the hypothesis that imatinib-loaded hNPs inhibit the suppressive effects of T-regs and hence protect tumor antigen presentation and cytotoxic T cells.

### Modulation of MDSCs

MDSCs are a group of heterogeneous immature myeloid cells that are generated in bone marrow and migrate to primary and metastatic tumors in response to cytokines or other immune mediators secreted by tumor cells [87]. Generally, two subsets of MDSCs have been identified in both humans and mice: polymorphonuclear MDSCs and monocytic MDSCs. Both subsets exert more potent nonspecific suppressive activities when recruited to tumor sites than their counterparts in peripheral lymphoid tissues, but the latter is believed to have a predominant role in T-cell suppression [88]. In the acidic and hypoxic TME, tumor-infiltrating MDSCs are driven to adopt FAO and OXPHOS as their main metabolic pathways [88, 89]. MDSCs also upregulate their expression of iNOS and arginase 1 and rapidly differentiate into TAMs [38]. MDSCs obstruct T-cell functions by depleting key nutrients such as arginine, cysteine and tryptophan (Trp) [88]. Furthermore, MDSCs have been linked to the induction of T-regs in the TME [90, 91].

Compelling evidence has demonstrated that, similar to TAMs, MDSCs also have M1 and M2 phenotypes [92, 93]. M1-like MDSCs are characterized as tumor-inhibiting cells by the production of TNF-α, IL-12, and nitric oxide, whereas M2-type MDSCs thwart the tumoricidal effect of T lymphocytes or NK cells by secreting TGF-β, IL-10 and arginase [94]. The repolarization of MDSCs may represent a new strategy to optimize current cancer immunotherapy. Several studies have shown that Toll-like receptor (TLR) signaling ligands hinder the immunosuppressive activity of MDSCs [95–97]. A study by Zhang et al. showed that cationic polymers, such as cationic dextran and polyethyleneimine, directly repolarize MDSCs and TAMs to the M1 phenotype via TLR4 signaling and promote the expression of Th1-inducing cytokines [98, 99]. Another study reported that synthetic high-density lipoprotein-based NPs have high affinity to scavenger receptor type B-1, which is expressed on MDSCs, and thus attenuate the immunosuppressive function of MDSCs [100]. However, further scrutinization may be needed to obtain more convincing corroboration for the interactions between these macromolecules and immune cells.

**Fig. 4:**
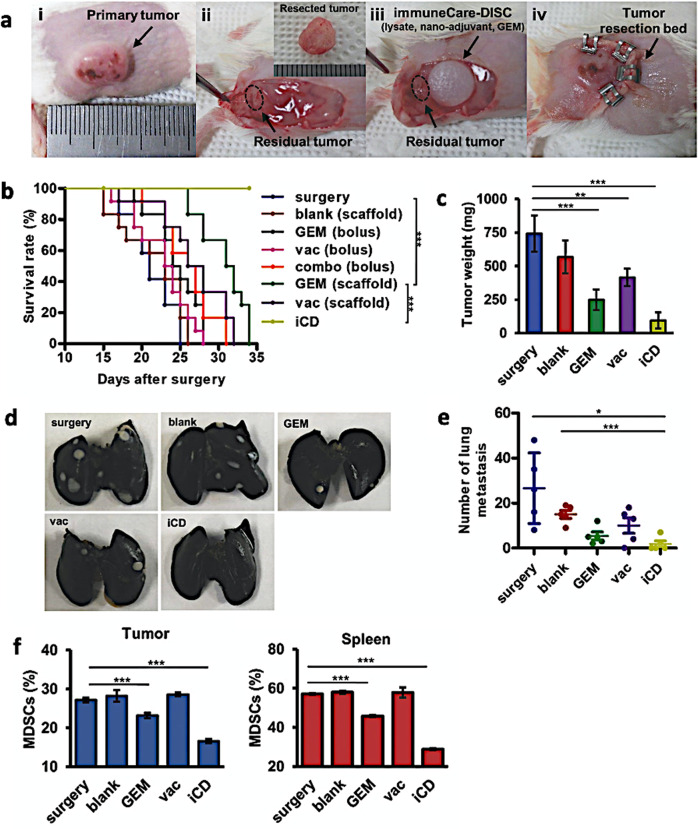
**a** Implantation approach: (i) Surgery was performed after the tumor volume reached about 300 mm^3^. (ii) Tumor dissection mimicking incomplete tumor removal (about 90% of primary tumor was excised). (iii) Implantation of the iCD containing GEM and cancer vaccines. (iv) Wound closure. **b** Survival rate of recurrent 4T1 tumor-bearing mice determined by log-rank test (*n* = 10). **c** Weight of recurring tumor on day 14 after surgery. **d** Representative images of lungs collected from mice in the different treatment groups at days 14 after tumor resection. White nodules indicate metastatic tumors in the lungs. **e** The mean numbers of macroscopically visible breast cancer metastases in the lungs. **f** FACS analysis demonstrating infiltrating MDSCs (CD11b^+^Gr1^+^) at day 7. Adapted from REF [103] with permission by WILEY-VCH.

Small molecular drugs also have potential to keep tumor-induced MDSCs at bay. Selective inhibition of MDSCs has been added to the portfolio of several well-known anticancer drugs, such as gemcitabine (GEM) [101], curcumin [102], and docetaxel [92], yet the mechanisms still need further investigation. Phuengkham et al. developed a hydrogel depot (iCD) containing tumor lysate, poly (I:C) in nanogel and GEM to revert the immunosuppressive TME and prevent postsurgical tumor recurrence and metastasis (Fig. 4) [103]. The peritumorally implanted scaffold prevented local recurrence of advanced 4T1 breast tumors and possible metastasis to the lungs, which were attributed to the synergy between MDSC-depleting GEM and vaccine-induced antitumor immunity. Sunitinib is an FDA-approved tyrosine kinase receptor (TKR) inhibitor for cancer chemotherapy. It has been proven to be a potent immunomodulator because it also targets TKRs expressed on MDSCs and thus depletes MDSCs in the circulation, spleen, and tumor [104–106]. Sunitinib could be encapsulated in polymeric micelles and utilized as an auxiliary treatment for cancer vaccines against advanced melanoma [107]. Antitumor immunity was strengthened by a reduced number of MDSCs and T-regs and increased influx of cytotoxic T cells and Th1 cytokine profiles when tumors were pretreated with micellar sunitinib. The same research team published a similar study with NPs loaded with CDDO-Me, a synthetic oleanane triterpenoid with anticancer properties, which augmented the vaccine-induced T-cell response against melanoma by blocking the function of MDSCs [108].

### Modulation of TAMs

**Fig. 5:**
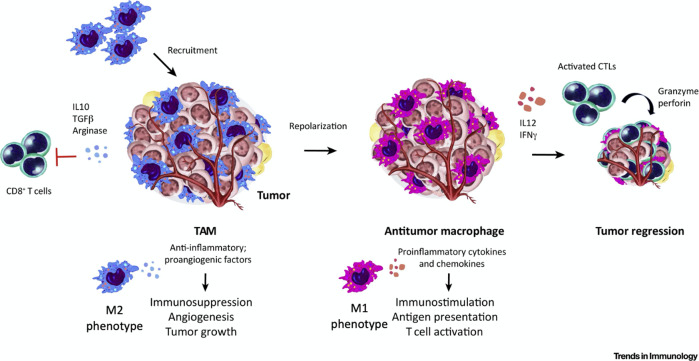
Macrophages accumulate in tumors by proliferation from tissue resident precursors or by trafficking from bone marrow-derived precursors. Once in tumors, these cells can adopt a tumor-promoting phenotype (M2) that induces immunosuppression, angiogenesis, tumor growth, and metastasis. Strategies to improve cancer therapies are being tested and include (i) blocking the recruitment of TAMs; (ii) inducing the repolarization of TAMs into an immunostimulatory phenotype (M1); and (iii) upregulating antigen presentation machinery that can activate CTLs, which can then lyse malignant cells to suppress tumor growth. Adapted from REF [111] with permission by Elsevier Ltd.

TAMs constitute a significant portion of cell populations in the TME and serve as major tumor-promoting immune cells, in addition to T-regs and MDSCs [109]. Macrophages with the M1 phenotype have cytotoxicity and upregulate the production of proinflammatory cytokines. However, M2-like TAMs secrete immunosuppressive cytokines, including IL-10 and TGF-β, which contribute to tumor progression and resistance to chemotherapies in part by inhibiting MHC-mediated antigen presentation and stimulating apoptosis of lymphocytes (Fig. 5) [110, 111]. Moreover, the density of TAMs is associated with poor long-term survival, increased angiogenesis and metastasis [112, 113]. Therefore, the ratio of M2 to M1 phenotype macrophages within tumors is a determinant of cancer immunotherapy success. In recent efforts, TAM depletion has been achieved by lipid NP-encapsulated siRNA silencing in inflammatory monocytes [114]. The technological advantages of biocompatible NPs in siRNA delivery, which are capable of systemic delivery to immune cells with nuclease stability and reduced immunostimulation, were also demonstrated. Furthermore, TAMs can be converted to the proimmunogenic M1 phenotype due to their plasticity. One study reported polarizing the protumorigenic M2 phenotype toward the antitumorigenic M1 phenotype using TLR agonist-loaded NPs. R848, an agonist of TLR7/8, was used as a potent driver of the conversion of the M2 to M1 phenotype and loaded into β-cyclodextrin NPs (CDNP-R848) [115]. The administration of CDNP-R848 modulated the tumor-supportive M2-like phenotype to its tumoricidal M1 counterpart, yielding improved immunotherapy response rates when combined with ICB and anti-PD-1 compared with the effects of ICB alone. These findings indicate the ability of drug-encapsulated NPs to efficiently modulate TAMs for cancer immunotherapy.

**Fig. 6:**
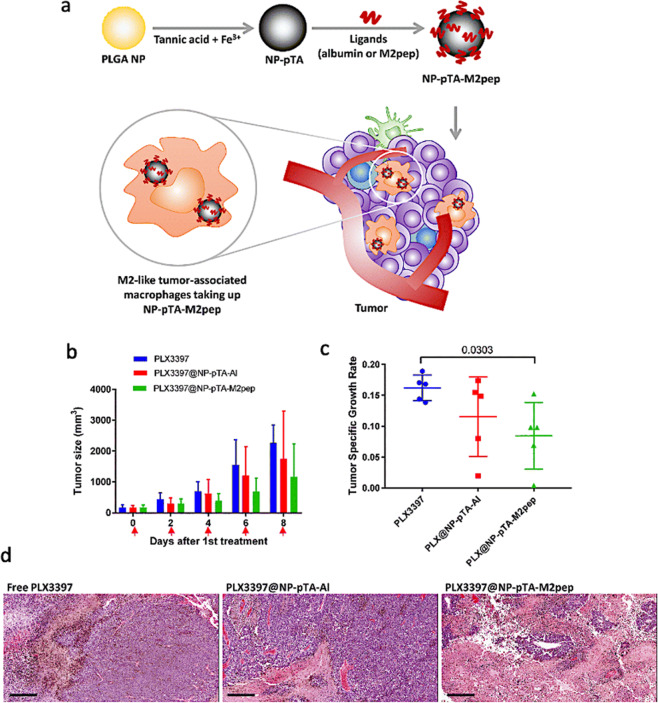
**a** Schematic illustration of M2pep-coated NPs and their interaction with TAMs in tumor. M2pep was conjugated to PLGA NPs via a simple surface modification method based on tannic acid-iron complex. **b** Tumor volumes recorded every other day. *n* = 5/group. **c** Specific growth rate of B16F10 tumor. ΔlogV/Δt (V: tumor volumes; t: time in days). *p*-values by Uncorrected Fisher’s LSD. **d** Histopathologic features of the tumor parenchyma treated with free PLX3397, PLX3397@NP-pTA-Al, or PLX3397@NP-pTA- M2pep. Scale bars: 300 μm: (left) free PLX3397-treated tumor composed of sheets of neoplastic epithelial cells with scattered foci of necrosis and hemorrhage; (center) PLX3397@NP-pTA-Al-treated tumor composed of neoplastic epithelial cells with a central core of necrosis expanded by fibroblasts, fibrin, and hemorrhage; (right) PLX3397@NP-pTA-M2pep-treated tumor composed of coalescing bands of necrosis composed of eosinophilic fibrillar material, erythrocytes, and a mixed inflammatory population. Adapted from REF [119] with permission by Springer Nature.

Effective TAM targeting is a major challenge due to a lack of high avidity and selectivity, although modulating the TME with therapeutics that deplete/reprogram TAMs has shown considerable potential. TAM targeting has been reported using NPs to improve the delivery of drugs. Small molecules, including mannose and folate, which are ligands for mannose receptor (CD206) and folate receptor β expressed by activated M2-like macrophages, have been used as NP surface modifiers to target TAMs [116–118] and have shown enhanced cellular uptake. To increase the selectivity of TAM targeting, Yeo et al. developed polymeric NPs coated with M2pep (YEQDPWGVKWWY) that preferentially binds to murine M2-like TAMs via an adhesive layer of tannic acid-Fe^3+^ complex (pTA) on the NP surface (Fig. 6) [119]. M2pep-coated NP-pTA (NP-pTA-M2pep) showed increased cellular uptake by M2-polarized bone marrow-derived macrophages in vitro and CD206^+^ macrophages in B16F10 melanoma in vivo compared with that of uncoated-NPs, indicating enhanced binding affinity of M2pep-coated NPs. This group also demonstrated that tumor growth was attenuated by drug-loaded NP-pTA-M2pep more than free drug in a mouse B16F10 melanoma model. These studies demonstrate that coating the NP surface with M2-like macrophage-targeting ligands facilitates the efficient delivery of drugs to TAMs with minimal effects on tumor cells.

### Modulation of ECM/tumor-associated fibroblasts (TAFs)

Tumors comprise a variety of cell populations, including proliferating cancer cells, immune-stimulating/immunosuppressive cells, endothelial cells, and perivascular cells, which are embedded within a protein-rich ECM. The ECM is often dense and stiff and acts as a physical and biochemical barrier to the transport of nanomedicines. The composition and structure of the ECM not only decelerate the movement of nanomedicines but also reduce the activities of therapeutic agents. Moreover, the dense and stiff ECM increases IFP and affects the transcapillary transport and diffusion of nanomedicines in the tumor interstitium [120, 121], limiting the distribution of nanocarriers to the vicinity of perivascular regions [122]. Approaches have been proposed to overcome these challenges by enhancing the intratumoral distribution of nanomedicines. Pretreatment of tumors with hyaluronidase has been shown to degrade the ECM and induce a transcapillary pressure gradient, thereby increasing the tumor uptake of liposomal doxorubicin (DOX) [123]. Priming tumors with an apoptotic inducer was used to reduce cell density, leading to enhanced drug penetration into solid tumors [124, 125]. Jessie et al. showed that apoptosis-inducing tumor priming with PTX-loaded polymeric microparticles expanded the interstitial space and resulted in a PTX concentration in tumors that was 16 times higher than the PTX/Cremophor EL (polyethoxylated castor oil) formulation and had lower toxicity [126]. In another study, Todd et al. reported that PEG surface coating of 145 nm radius superparamagnetic NPs improved NP transport and biodistribution by reducing nonspecific adhesion of NPs to the ECM [127].

Fibroblasts are transformed to TAFs by cytokine signaling in tumors [128], such as TGF-β and IL-10 [129], which results in cancer progression [130] and hinders tumor-specific immunity [131, 132]. TAFs are abundant in desmoplastic tumors and generate large quantities of ECM proteins, forming barriers that inhibit the transport of NPs [133]. Therefore, various types of NPs have been designed for TAF depletion to improve the interstitial transport and distribution of nanomedicines. For example, Huang et al. developed lipid/calcium/phosphate (LCP) NPs to facilitate improved accumulation and metabolic stability of quercetin, which suppresses the expression of Wnt16, a protein that regulates the induction of apoptosis and inhibits angiogenesis and proliferation in TAFs [134]. Quercetin prodrug-loaded LCP NPs (LCP-QP) with an average size of 35 nm showed greater NP penetration and tumor inhibition effects in a stroma-rich bladder carcinoma model compared with those of the parent quercetin due to a decrease in the active fibroblast population and collagen deposition in the TME. In addition, this group used similar targeted NPs with TNF-related apoptosis-inducing ligand (sTRAIL), which causes apoptosis in tumor cells, to modulate TAFs via the TGF-β signaling pathway to treat desmoplastic cancers [135]. sTRAIL-loaded LCP NPs induced TAF inactivation and resulted in retarded tumor growth. Collectively, modulating ECM/TAFs by engineered NPs is a promising strategy to favorably reduce transport barriers to drug delivery.

## Modulation of enzymes/cytokines in the TME

### Modulation of enzymes

ECM-associated enzymes are elevated in the TME and are responsible for cell proliferation and differentiation, contributing to ECM stiffness and degradation [136, 137]. Matrix metalloproteases (MMPs), such as MMP-2 and MMP-9, are expressed by cancer cells and stromal cells. They exert their proteolytic activity to break down the ECM and facilitate angiogenesis, which leads to tumor progression and metastasis [136]. Accordingly, MMPs have been investigated for preventing and treating tumors; however, the delivery of MMP inhibitors has been challenging due to poor specificity. Recent studies have shown the use of NPs with potential MMP inhibitors to control metastasis in cancer therapy [138, 139]. For example, Wang et al. created copper monosulfide nanocrystals modified with mesoporous silica and PEGylation (CuS@mSiO_2_-PEG). They found a reduction in the metastasis of cancer cells and improved survival rates after subcutaneous injections of HeLa cells that were prestimulated with Cus@mSiO_2_-PEG NPs in comparison with those of cells that were prestimulated with free copper in a HeLa lung metastasis model [138], demonstrating that NPs enhance therapeutic efficacy. Further studies have focused on a new target enzyme candidate, lysyl oxidase (LOX), which is elevated in the TME. LOX catalyzes crosslinking of collagen and elastin, which promotes stiffness of the ECM and malignancy [140, 141]. One study reported coating PLGA NPs with LOX inhibitory antibody (LOX_Ab_) through carbodiimide chemistry (LOX_Ab_NPs) to target LOX and manipulate collagen crosslinking by modulating LOX expression and/or activity [142]. The LOX_Ab_NPs suppressed 4T1 tumor growth in mice to a greater extent than LOX_Ab_ alone at the same dose (50 μg LOX_Ab_/kg), inhibited collagen crosslinking and suppressed ECM fibrosis in vivo. These findings also suggest that the NP formulation could achieve therapeutic efficacy by reducing the dose of soluble antibodies, thereby minimizing systemic side effects.

To restore the viability of T cells in the TME, a study aimed to silence lactate dehydrogenase A (LDHA), an enzyme expressed by tumor cells that catalyzes the conversion of pyruvate to lactate, contributing to acidic pH and T-cell anergy in the TME [143]. LDHA-silencing RNAs were systemically delivered by cationic lipid NPs. This siRNA treatment efficiently downregulated LDHA levels and thus attenuated lactate accumulation and neutralized the pH in the TME. When combined with anti-PD1 therapy, this strategy significantly delayed tumor progression in 4T1 and B16 melanoma tumor models with a substantial increase in infiltrated antitumor CD8^+^/CD4^+^ T cells and NK cells and a reduced population of T-regs in the TME.

Alternatively, inhibitors of IDO have been utilized in cancer therapy to offset immunomodulatory actions in the TME. IDO, an intracellular monomeric and heme-containing enzyme, starves the TME of Trp and increases the Trp-derived metabolite kynurenine pathway, causing immunosuppressive effects [144]. Many efforts have focused on IDO blockade treatment in combination with other immune checkpoint inhibitors to further augment antitumor activities and survival. Nie et al. developed a peptide assembling NP for the codelivery of a short d-peptide antagonist of programmed cell death-ligand-1 (^D^PPA-1) that binds PD-L1 and NLG919, a highly selective inhibitor of IDO [145]. By encapsulating NLG919 in NPs, this dual-targeted immunotherapeutic NP, denoted NLG919@DEAP-^D^PPA-1, improved the bioavailability of NLG919 and thus reduced dose-dependent toxicity. Furthermore, when injected IV in a murine model of melanoma, delayed tumor growth and extended mouse survival were observed in the NLG919@DEAP-^D^PPA-1 group to a greater extent than the groups treated with free NLG919 and NLG919-encapsulated DEAP NPs containing a scrambled amino acid sequence instead of the ^D^PPA-1 sequence. Another strategy involves a chemo drug, such as DOX, which can simultaneously trigger immunogenic cell death (ICD) using self-assembled liposomes composed of a phospholipid-conjugated prodrug form of indoximod (IND), an IDO inhibitor [146]. The DOX-encapsulated and IND-conjugated liposomes (DOX/IND liposomes) induce ICD and increase phosphorylation of S6 kinase in the mTOR pathway, reversing the immunosuppressive activity of IDO; furthermore, the pharmacokinetics of DOX or IND were improved in the 4T1 tumor model, demonstrating the use of nanoliposomes to enhance the permeability and retention of drugs.

### Modulation of cytokines

Cytokines play an important role in modulating immune responses, and an imbalance between stimulatory and suppressive cytokines causes immunosuppressive effects in the TME [147]. Although cytokines have been considered potent modulating agents for immunotherapy, many cytokines are, unfortunately, unstable and have a short half-life in circulation, resulting in poor therapeutic effects and serious side effects if delivered systemically [148, 149]. For instance, IL-2 is an agent that is approved by the FDA for cancer immunotherapy of metastatic melanoma and renal cell carcinoma. Systemic administration of IL-2 involves the risk of adverse effects, including thrombocytopenia and lymphopenia [149]. Thus, efforts have been made to sustain and specifically deliver cytokines to tumors using NPs. One early study incorporated IL-2 into multilamellar liposome drug carriers to develop a tumor vaccine adjuvant and showed that intravenous injection of IL-2 liposomes decreased hematologic toxicities in rats, indicating that NPs are capable of reducing drug toxicity [150]. In addition, Egilmez et al. encapsulated IL-12 and/or granulocyte-macrophage colony-stimulating factor (GM-CSF) in polylactic acid microspheres to induce both innate and adaptive antitumor immune responses [151]. The combination of IL-12 and GM-CSF in microspheres was superior to treatment with either cytokine alone in enhancing antitumor immunity and long-term survival.

**Fig. 7:**
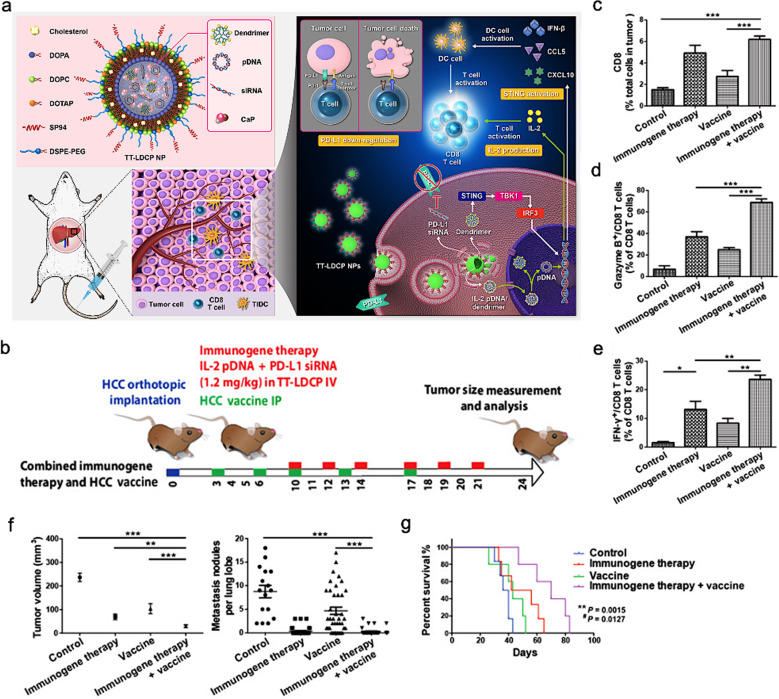
**a** ﻿Schematic representation of the mechanism of immunogene therapy by TT-LDCP NPs containing siRNA against the immune checkpoint PD-L1 and pDNA encoding the immunostimulating cytokine IL-2. Active tumor targeting was achieved through the addition of hepatocellular carcinoma (HCC)-targeted SP94 peptide to the surface of the NPs. The thymine-capped PAMAM dendrimer/CaP complexes achieved highly efficient gene transfection efficacy by enhancing nuclear delivery of the pDNA. Furthermore, thymine-capped PAMAM dendrimers stimulate the stimulator of interferon genes (STING) pathway and serve as an adjuvant to promote the maturation of intratumoral DCs. Efficient tumor-targeted codelivery of PD-L1 siRNA and IL-2 pDNA achieves tumor-specific expression of IL-2 and downregulation of PD-L1, increases infiltration and activation of CD8^+^ T cells in HCC, and induces a strong tumor-suppressive effect in HCC in synergy with a vaccine. CaP calcium phosphate, TIDC tumor-infiltrating dendritic cell, TT-LDCP NPs tumor-targeted lipid-dendrimer-calcium-phosphate NPs, IFN-γ interferon-γ. **b** ﻿Three days after the implantation of HCA-1 cells, mice were injected intraperitoneally five times (at 2- to 3-day intervals) with the HCC vaccine. For the combination groups, mice treated with the HCC vaccine received intravenous immunogene therapy (1.2 mg siRNA and pDNA/kg per dose) on days 10, 12, 14, 17, 19, and 21. Immunogene therapy: IL-2 pDNA and PD-L1 siRNA in TT-LDCP; vaccine: 5 × 10^6^ mitomycin C-treated cGM-CSF-overexpressing HCA-1 cells. **c** ﻿Combination of immunogene therapy and the vaccine increased the number of CD8^+^ T cells in tumors, as measured by flow cytometry (control, *n* = 18; immunogene therapy, *n* = 10; vaccine, *n* = 6; combination group, *n* = 6). Data are means ± SEM. ﻿**d** The immunofluorescence of granzyme B-positive CD8^+^ T cells in HCA-1 tumors was quantified 24 days after implantation for the treatment with immunogene therapy or the HCC vaccine. (control, *n* = 8; immunogene therapy, *n* = 6; vaccine, *n* = 6; combination group, *n* = 7). **e** IFN-γ intracellular staining in tumor-infiltrating CD8^+^ T cells measured by flow cytometry (*n* = 5). The combination of immunogene therapy and vaccine treatment significantly reduced tumor sizes (control, *n* = 12; immunogene therapy, *n* = 12; vaccine, *n* = 12; combination group, *n* = 24) and distal lung metastatic nodules (**f**) and increased the overall survival (**g**) (*n* = 5, **P < 0.01 compared with control; ^#^*P* < 0.05 compared with vaccine treatment) in an orthotopic HCC model. Adapted from REF [155] with permission by AAAS under Creative Commons Attribution-NonCommercial license.

Despite the promise of cytokine-based therapy in cancer treatment, systemic administration of cytokines remains a challenge due to potential systemic toxicity, limiting the efficacy and dose [152, 153]. To overcome this challenge, researchers have adapted NP-encapsulated cytokine-encoding plasmids to modulate cytokine levels in the TME. One group generated a self-assembled NP consisting of methoxy poly(ethylene glycol)-poly(lactide) (MPEG-PLA) and 1,2-dioleoyl-3-trimethylammonium-propan, named DMP, with a zeta-potential value of 38.5 mV and a size of 37.5 nm for delivery of the IL-12 plasmid (pIL12), which can activate innate and adaptive immunity [154]. The pIL12 and DMP complex significantly inhibited tumor growth by suppressing tumor angiogenesis, promoting apoptosis and reducing proliferation in both subcutaneous and peritoneal colon cancer models, with increased expression of IL-12 in the absence of noticeable adverse effects induced by IL-12, demonstrating the potential of cytokine gene delivery nanocarriers. Another interesting approach to improving anticancer efficacy and circumventing systemic toxicity was reported using tumor-targeted lipid-dendrimer-calcium-phosphate (TT-LDCP) NPs [155]. Chen et al. designed tumor-targeting lipid NPs to deliver siRNA against the immune checkpoint ligand PD-L1 and immunostimulatory IL-2-encoding plasmid DNA, which reprogrammed the TME and synergized with a hepatoma vaccine by increasing tumor-infiltrating CD8^+^ T cells and the expression of IFN-γ and granzyme B, resulting in significantly improved antitumor efficacy compared with that of vaccine alone (Fig. 7). These results demonstrated the potential of immune gene therapy to modulate the TME.

## Concluding remarks

The recent successes of several immunotherapeutic agents in the clinic have motivated the field to optimize current approaches to tackle unresolved issues, such as limited responses to immunotherapy [156], development of resistance [157], and toxicities associated with therapies including ICB, cytokine therapy and CAR-T cell infusion [158–160]. In the past several decades, cancer nanomedicine has confronted similar problems, and those experiences could be utilized to improve current immunotherapies. As we reviewed here, nanoenabled delivery systems can reprogram the TME and thus hold great promise to amplify antitumor immune responses.

Immune context in the TME is the medium in which immunotherapies struggle to triumph. Controversies still exist regarding the influence of altered metabolism on the development and functions of infiltrated immune cells [88, 161]. Several metabolic targets in the TME and ligands have been identified; however, optimal delivery strategies still need further development [10, 162]. In addition to the aforementioned immune cells, the functions of tumor-infiltrated B cells remain inadequately explored. Clinical data indicate that the enrichment of CD20^+^ B cells in the TME is associated with improved immunotherapeutic responses and survival in patients with melanoma, sarcoma, or renal cell carcinoma who received ICB [163–165]. In addition, accumulating clinical evidence has demonstrated the correlation between increased density of intratumoral tertiary lymphoid structures (TLSs) and the positive responses of patients to chemo- or immunotherapies, suggesting that TLS formation could be exploited as a prognostic indicator of tumor sensitivity to treatments [164]. Studies have also proven that TLSs serve as shelters for immune cells under attack by the TME and can support the antigen presentation and education of infiltrated T and B cells and hence enhance antitumor immunity [166]. The importance of TLSs has been recognized, and strategies that promote TLS neogenesis may represent a promising direction for cancer therapy.
